# The effect of different government subsidies on total-factor productivity: Evidence from private listed manufacturing enterprises in China

**DOI:** 10.1371/journal.pone.0263018

**Published:** 2022-01-31

**Authors:** Dongmei Wang, Yangyang Sun

**Affiliations:** 1 School of Economics, Zhejiang Gongshang University, Hangzhou, China; 2 Faculty of Business Administration, School of Business Administration, Southwestern University of Finance and Economics, Chengdu, China; Szechenyi Istvan University: Szechenyi Istvan Egyetem, HUNGARY

## Abstract

Private enterprises play an increasingly important role in China. They can improve the total-factor productivity (TFP) and help transform and upgrade industrial structures. This study uses data for private listed manufacturing companies from 2009 to 2017 to examine the effects of different types of subsidies on TFP. We also analyze the heterogeneity and specific mechanism of subsidy effects. We find that R&D subsidies and production subsidies positively affect private enterprises’ TFP. Moreover, R&D subsidies and production subsidies lagged by one period can also significantly increase private enterprises’ TFP. In terms of industry, R&D subsidies have more obvious effects on technology-intensive industries, while production subsidies have more significant effects on labor-intensive and capital-intensive industries. In terms of scale, R&D subsidies’ effects on the TFP of medium-sized enterprises are the largest, while production subsidies have the greatest effect on small enterprises’ TFP. Government subsidies increase private enterprises’ TFP through two mechanisms: improving technological innovation capability and alleviating financing constraints. Our results suggest that governments should formulate different subsidy policies according to industry and enterprise scale.

## 1. Introduction

China’s rapid economic growth has had serious consequences in terms of resource consumption and environmental impact. In this context, innovative green development has become a major direction for socioeconomic growth. It is necessary, therefore, to give full play to the role of innovation in leading economic development. Private enterprises play an important role in such innovation. For example, 80% of patent applications are made by private enterprises, among which more than 60% are invention patents, while new-product provision accounts for about 70% [1]. Meanwhile, the level of total-factor productivity (TFP) largely depends on innovation capability. Therefore, private enterprises also play an important role in improving TFP. In China, the overall TFP of enterprises is low and relatively slow [2–4]. Improving the productivity and innovation of private enterprises is therefore important and is a topic of concern to both researchers and the government.

Because of limitations in terms of credit funds, production technologies, and innovation externalities, many firms lack the driving force needed to improve TFP [5,6]. Government subsidies can help overcome this by promoting technological progress and economic growth. As a traditional intervention policy, subsidies can compensate for market failures and promote the rapid development of industries. Government subsidies have been widely implemented not only in China but also in Europe and the United States [7].

Previous studies have mainly investigated the effects of government subsidies on enterprises’ TFP in terms of financing constraints, innovation incentives, and signaling effects [6,8,9]. Several studies have reported that government subsidies can increase enterprises’ TFP by increasing enterprise liquidity and reducing fixed or marginal costs [5,6,10]. Others, by contrast, have reported negative effects, revealing that companies might use subsidies for projects other than R&D investment, thus making government subsidies likely to inhibit TFP [11–13]. Another possible reason for the negative effects of subsidies on TFP pertains to government motives, which may be distorted. Enterprises with government connections are often more likely to receive government subsidies; this type of selection bias can reduce the positive effects of subsidies [14]. Rent seeking and corruption can also distort government subsidies, leading to a loss of subsidy efficiency. Finally, some studies have found a nonlinear relationship between government subsidies and corporate TFP. Government subsidies can promote enterprises’ TFP within a certain range, but there is no effect, or even a restraining effect on TFP when subsidies are beyond a reasonable range [15–17]. Overall, there are contradictory findings regarding subsidy effects, which may be attributable to factors such as the various definitions of government subsidies, measurement methods for TFP, or differences in sample selection.

Governments formulate different subsidy policies based on different policy objectives. The effects of these different subsidy policies on enterprises’ technological innovation will be significantly different and will therefore have different effects on TFP. However, previous studies have not examined subsidies’ effects on TFP in terms of different subsidy methods, which is a limitation of the existing literature. Previous studies have also neglected to demonstrate the effects of different subsidies on enterprises’ TFP.

The present study, therefore, subdivides government subsidies into R&D subsidies and production subsidies. Using a sample of private listed companies in China, we examine the overall effects and heterogenous effects of different subsidies on enterprises’ TFP and further explore the mechanisms of different subsidies. This study contributes to the literature in three aspects. First, we divide government subsidies into R&D and production subsidies and study their effects on private enterprises’ TFP. Second, we conduct heterogeneity analyses at the industry and scale levels, which can provide a reference for evaluating the effects of government subsidy policies. Third, we select private listed manufacturing companies as the sample, reflecting a degree of novelty in sample selection.

This paper is organized as follows. Section 2 sets out the theoretical analysis and research hypotheses. Section 3 describes the data, variables, and empirical approach, while Section 4 presents the results and robustness tests. Section 5 concludes and discusses the policy implications.

## 2. Theoretical analysis and hypothesis development

### 2.1 R&D subsidies, technological innovation, and private enterprises’ TFP

Technological innovation has diffusion and spillover effects, which can have positive effects on enterprises’ TFP [18,19]. Specifically, technological innovation can change the proportion of internal factor inputs in an enterprise, causing labor prices to rise and capital prices to fall. Therefore, enterprises will introduce advanced equipment to expand the scale of equipment investment and then use economies of scale to increase TFP. It has been noted that technological innovation can help improve resource allocation efficiency, thereby increasing the level of TFP [3]. However, technological innovation carries certain risks and uncertainties for enterprises. Government subsidies can compensate for externalities in enterprise innovation activities to some extent [20,21]. Yan and Wu [22], for example, found that government subsidies have significant positive effects on both substantive and strategic innovation. Specifically, government subsidies can improve the investment efficiency of R&D activities and TFP by providing enterprises with sufficient R&D funds and encouraging them to invest in innovative projects. Government subsidies not only reduce enterprises fixed and marginal costs but also promote innovation and TFP [5]. Liu and Zhao [23] found that government subsidy policies can promote the technological progress of new-energy vehicle companies. Carboni [24] found that government subsidies, especially R&D subsidies, can increase enterprises’ R&D investment and thus enhance their productivity. Meng et al. [25], however, found that companies will increase R&D investment and enhance their innovation capabilities only when they receive sufficient product subsidies.

Government subsidies can also increase enterprises’ TFP by improving their innovation efficiency. Given the spillover effects of innovation activities, the private benefits of innovation activities are lower than social benefits, leading to a problem of insufficient investment in corporate innovation. Government subsidies, however, can improve the investment efficiency of corporate innovation activities and increase TFP [26].

Meanwhile, R&D subsidies can promote TFP by improving operating efficiency, knowledge stock, and resource allocation efficiency. As the most direct way to support and guide industrial development, government subsidies are useful for improving business efficiency. They provide an important guarantee for improving operational efficiency [27]. By investigating the effect of outgoing officials’ accountability audits of natural-resource assets on corporate innovation, Liu et al. [28] found that government subsidies can improve innovation investment efficiency and technological innovation levels. On the one hand, R&D subsidies can increase a company’s knowledge stock and promote the adoption of new technologies and the upgrading of old technologies [29,30]. On the other hand, R&D subsidies can alleviate underinvestment in corporate innovation projects, improve the scale of enterprise R&D investment, and improve the efficiency of resource allocation [31,32].

R&D subsidies mainly support enterprises’ technological innovation activities and provide obvious incentives for such activities. Accordingly, the following hypothesis is proposed:

**Hypothesis 1**: R&D subsidies increase private enterprises’ TFP by promoting technological innovation.

### 2.2 Production subsidies, financing constraints, and private enterprises’ TFP

Financing constraints limit the allocation of corporate resources and inhibit the scale of corporate investment, which will limit TFP growth [33,34]. Government subsidies can alleviate these financing constraints and help increase TFP [35]. Montmartin and Herrera [36] noted that government subsidies can reduce enterprises’ transaction costs and fixed costs, thereby reducing their financing costs, improving their innovation capabilities and TFP. First, government subsidies provide direct financial support for enterprises, which will alleviate internal financing constraints and promote the development of economies of scale [37]. Grilli [38] found that government subsidies can help increase enterprises’ financing channels, reduce the pressure of financing constraints, and promote technological innovation and productivity improvement. Government subsidies also provide an implicit guarantee for enterprises’ external financing, which sends a signal that an enterprise is developing well. This can enhance investor confidence and relieve external financing constraints to a certain extent [39]. Moreover, governments often grant subsidies to companies with higher innovation levels and better development prospects, which sends an economic signal to banks and other financial institutions. Subsidies can therefore broaden firms’ financing channels and improve their investment efficiency. In addition, government subsidies can provide a certain price compensation for firms, which is conducive to forming price advantages and incentives for innovation in corporate management [40]. Finally, government subsidies can reduce enterprises’ transaction costs and liquidity risks and thus promote innovation efficiency [41].

Compared with other types of enterprises, private enterprises face serious financing obstacles in China, and such constraints negatively affect TFP [42]. Government production subsidies are mainly intended for enterprises’ product-related activities (e.g., expanding production and markets). In this way, production subsidies can help improve enterprise investment efficiency and scale efficiency, thereby improving TFP [43]. Therefore, the following hypothesis is proposed:

**Hypothesis 2:** Production subsidies increase the level of private enterprises’ TFP by alleviating financing constraints.

Based on the above theoretical analysis, we found that the effects of R&D subsidies and production subsidies on TFP, as well as the specific mechanisms of action, are quite different. However, previous studies have paid insufficient attention to the effects of different types of subsidies on enterprises’ TFP. In addition, many studies only focus on the effect of government subsidies on overall enterprise technological innovation and TFP, without considering private enterprises as a separate research object. However, the role of private enterprises in China’s economic development has become increasingly important, and it is therefore important to explore the effect of government subsidies on private enterprises’ TFP. Finally, different types of enterprises are affected in different ways by different subsidy policies, which may account for inconsistencies in the research findings. Clarifying these issues is therefore of great importance for improving the effects of subsidy policies and promoting enterprise production efficiency. This study, therefore, uses a sample of Chinese private listed manufacturing companies covering 2009–2017 to investigate the effects of different types of government subsidies on enterprises’ TFP and explore the mechanisms of different subsidies. Finally, we analyzed the heterogeneous responses of different types of enterprises to subsidy policies. This study provides a new research perspective for evaluating government subsidy policies. On the one hand, it can enrich and expand research on government subsidies and enterprises’ TFP; on the other hand, it can provide policy implications for improving the effectiveness of government subsidy policies and increasing enterprises’ TFP.

## 3. Data, empirical approach, and variables

### 3.1 Data

A-share private listed manufacturing enterprises in China were selected as the research sample. We selected 2009–2017 as the sample period, mainly for the following reasons: First, before 2009, private listed companies accounted for a small proportion of Chinese firms and are not representative. Including them in the analysis could result in biased, unrepresentative research conclusions. Second, before 2009, there were missing values for many key variables for private listed companies. For example, the lack of intermediate input can cause problems in calculating TFP, and the lack of data on R&D intensity would make model estimation impossible. Further, insufficient data for company size, net profits, and time to list would make it impossible to calculate the relevant control variables. If those variables are not controlled for, the obtained policy effects will not be credible. Third, there is a great deal of missing government subsidy data and enterprise-level patent data for 2018–2020, making it difficult to estimate the effects of recent subsidy policies.

The industry classifications used in this study follow the 2012 China Securities Regulatory Commission’s classifications. Government subsidy data and enterprise-level data come from the China Stock Market & Accounting Research Database. Macrolevel data are from the 2010–2018 China Statistical Yearbooks and Provincial and Municipal Statistical Yearbooks. Based on the literature, we processed the company data as follows: (1) exclude special treatment (ST)-share companies; (2) exclude companies listed after 2015 (due to a lack of data); (3) exclude companies with zero employees; (4) exclude companies with negative net fixed assets, total industrial output, and other indicators; and (5) exclude companies in Tibet (due to lack of data). Finally, 874 firms and 7866 observations were obtained for analysis (see S1 Dataset for details).

### 3.2 Empirical approach

Our research sample has a standard panel data structure that contains two dimensions: cross-firm and time dimensions. To reduce the effect of data fluctuations and make the result more accurate, we take the logarithms of R&D subsidies, production subsidies, TFP, R&D expenditures, and patent variables. The model is constructed as follows:

$$lnTFP_{it}=\beta_{0}+\beta_{1}lnRD_{it}+\beta_{2}lnSub\_rd_{it}+\beta_{3}lnSub\_rd_{it}*lnRD_{it}+\sum_{j=1}^{7}\;\delta_{j}\;X_{jit}+u_{i}+v_{t}+\varepsilon_{it},$$ (1)

$$lnTFP_{it}=\theta_{0}+\theta_{1}lnPatent_{it}+\theta_{2}lnSub\_rd_{it}+\theta_{3}lnSub\_rd_{it}*lnPatent_{it}+\sum_{j=1}^{7}\;\delta_{j}\;X_{jit}+u_{i}+v_{t}+\varepsilon_{it},$$ (2)

$$lnTFP_{it}=\gamma_{0}+\gamma_{1}CF_{it}+\gamma_{2}lnSub\_cf_{it}+\gamma_{3}lnSub\_cf_{it}*CF_{it}+\sum_{j=1}^{7}\;\delta_{j}\;X_{jit}+u_{i}+v_{t}+\varepsilon_{it},$$ (3)

where the dependent variable, TFP_it_, is the TFP of firm i during year t; lnsub_rd_it_ and lnsub_cf_it_ are the logarithm of the number of R&D and production subsidies, respectively, which are the core explanatory variables of interest in this study. The mediator variables are RD_it_, Patent_it_, and CF_it_, denoting the levels of innovation input, output, and financing constraints, respectively. X_jit_ is a set of control variables. When δ = 1, 2, 3, 4, it is a control variable at the firm level; when δ = 5, 6, 7, it is a control variable at the regional level. u_i_ and v_t_ refer to firm-fixed and time-fixed effects, respectively, and ε_it_ is the error term. These coefficients capture the effects of the independent variables on TFP. Models (1) and (2) examine the effect of government R&D subsidies on TFP after adding the variables of innovation input and output. Model (3) analyzes the effect of production subsidies on TFP after adding the mechanism variables of financing constraints.

### 3.3 Variables

#### 3.3.1 Dependent variable

Total-factor productivity (TFP). The main methods for measuring an enterprise’s TFP are DEA-Malmquist, OLS, OP [44], and LP [4,45,46]. In the DEA-Malmquist index method, samples cannot be randomly selected, and the measurement results will be affected by random errors. Likewise, OLS will produce sample-selectivity bias and simultaneity bias. Moreover, the OP method cannot estimate samples whose investment amount is zero or missing, leading to a loss of estimated samples. For these reasons, we selected the LP method to measure enterprises’ TFP.

Based on the C-D function, the introduction of intermediate input factors sets the TFP model as

$$lnY_{it}=\beta_{0}+\beta_{l}lnL_{it}+\beta_{k}lnK_{it}+\beta_{m}lnM_{it}+\varepsilon_{it},$$ (4)

$$TFP=lnY_{it}-\beta_{l}lnL_{it}-\beta_{k}lnK_{it}-\beta_{m}lnM_{it},$$ (5)

where Y and L are the main business income (million dollars) and the number of workers, respectively. K and M denote the net fixed assets (million dollars) and the amount of purchased goods and services (million dollars), respectively. ε_it_ is the random error term, and subscripts i and t represent the firm and year, respectively.

#### 3.3.2 Independent variable

The logarithm of the number of R&D and production subsidies (lnSub_rd and lnSub_cf). Based on intentions, government subsidies are divided into production subsidies and R&D subsidies. Referring to the literature, we used the keyword method to search for the specific project name in the government subsidy details [47]. Further, we distinguished R&D subsidies and production subsidies from the total subsides. R&D subsidies are subsidies that use technology, new products, R&D, and so on. Production subsidies are subsidies that use investment promotion, job stabilization, market development, and so on. S1 Table presents the specific classification methods.

#### 3.3.3 Mechanism variables

(1) enterprise innovation (R&D and Patent). These two variables have been widely used to measure enterprise innovation. In this study, innovation input is measured by enterprise R&D expenditure, while innovation output is measured by the total number of patents granted for utility, invention, and appearance. (2) Financing constraints (CF). Existing studies have used multiple indicators to measure financing constraints, such as the KZ index [48], WW index [49], and SA index [50]. The KZ and WW indexes have endogeneity problems [51]. To avoid such problems, we used the SA index to measure corporate financing constraints [50]. The formula for calculating the SA indicator is SA = −0.737 × Size + 0.043 × Size^2^–0.04 × Age, where Size is the size of the company (measured by the logarithm of net fixed assets in millions of dollars), and Age is the age of the company. Since SA is a negative value, when the value is smaller, the financing constraints faced by the enterprise are more severe.

#### 3.3.4 Covariates

Following existing TFP research [52,53], we set the covariates as follows: Enterprise-level covariates include Enterprise size (Size), Enterprise age (Age), Capital structure (ALR), and Asset liquidity (Ass_liq). Regional-level covariates include Human capital (Per), Foreign investment (FDI), and Economic development (Per_gdp). Table 1 summarizes the names and definitions of the variables used in this study.

**Table 1:** List of variables and descriptive statistics (observations = 7866).

Variables	Definition	Mean	SD	Min	Max
lnTFP	Calculated based on LP method	1.408	0.211	−1.618	2.053
lnSub_rd	Logarithmic value of R&D subsidy	1.090	1.599	−7.824	14.365
lnSub_cf	Logarithmic value of production subsidies	0.917	1.858	−9.045	16.819
lnRD	Logarithmic value of R&D expenses	3.436	1.361	−5.473	9.048
lnPatent	Logarithmic value of total granted number of patents	2.728	1.370	0	0.527
CF	SA Index	−3.021	0.471	−4.157	−0.258
Size	The logarithm of net fixed assets	6.256	5.610	0	27
Age	Observation year-listing date +1 (year)	5.882	1.335	0.301	10.675
Ass_liq	1-Ratio of total liabilities to total asset (%)	37.199	20.285	0.708	391.454
ALR	The ratio of total liabilities to total assets (%)	0.285	0.259	−2.800	0.972
Per	The proportion of students in universities (%)	1.880	0.401	0.680	3.350
FDI	Amount of foreign direct investment (10000 dollars)	1495388	914879.900	1383.500	3575956
Per_gdp	GDP per capita (CNY/Person)	59530.090	23423.120	10971	128994.100

Note: SD, standard deviation; Min, minimum; Max, maximum; All data processing and empirical analysis in our research are operated by Stata15 software.

## 4. Empirical analysis

### 4.1 Descriptive statistics

Table 1 presents the statistical characteristics. We can see that the minimum value of TFP is negative, which means that the TFP of some private enterprises has declined. The standard deviation of government subsidies is too large, which means that the number of subsidies received by private enterprises differs considerably. When implementing a subsidy policy, the government will make allocations according to the innovation levels of enterprises.

### 4.2 Basic model results

The coefficients in columns (1) and (2) of Table 2 are 0.005 and 0.008, respectively, with a significance level of 1%. This indicates that R&D subsidies and production subsidies have significant effects on TFP. Comparing the regression coefficients of the two subsidies, we found that the effect coefficient of R&D subsidies is slightly lower than that of production subsidies. Therefore, supporting private enterprises’ production subsidies to alleviate their financing difficulties may have a more direct stimulating effect on TFP. The control variables included in our model can help explain the variations in TFP. Enterprise size, asset liquidity, debt-to-asset ratio, and asset-liability ratio all have a positive effect on private enterprises’ TFP, and all are significant at least at the 1% level. Regional economic development and human capital significantly affect private enterprises’ TFP. This shows that the higher the level of economic development and the higher the quality of human capital, the larger the effect on TFP. Although enterprise age and foreign investment lack statistical significance, their effect on private enterprises’ TFP is consistent with expectation. The results in Table 2 clearly show that different types of government subsidies have significant effects on private enterprises’ TFP. We also found that the effect of production subsidies is larger than that of R&D subsidies.

**Table 2:** Estimated effects of government subsidies on TFP.

	lnTFP	
Variables	(1)	(2)
lnSub_rd	0.005***	
	(4.22)	
lnSub_cf		0.008***
		(5.91)
Age	0.003	0.001
	(0.60)	(0.20)
Ass_liq	0.269***	0.238***
	(5.72)	(5.03)
ALR	0.004***	0.003***
	(6.84)	(4.16)
Size	0.040***	0.039***
	(4.31)	(4.37)
Per_gdp	3.67e-07**	3.25e-07*
	(2.63)	(2.12)
Per	0.016**	0.007*
	(2.32)	(2.18)
FDI	1.32e-09	4.24e-10
	(0.34)	(0.12)
Constant	0.875***	0.932***
	(10.22)	(10.47)
Firm-fixed effect	Yes	Yes
Time-fixed effect	Yes	Yes
R^2^	0.364	0.373
Observations	7595	7633

Note: t statistics are reported in parentheses; *** p<0.01,

** p<0.05,

* p<0.1.

### 4.3 Effect of government subsidy lag period

There may be a certain lag in the effect of government subsidies, that is, the implementation effect of government subsidies will not appear in the current period, and a certain buffer period is required. Previous studies of subsidies’ effects on enterprises’ TFP have rarely examined such a lag effect. Accordingly, this study establishes a lag period model for government subsidies to better analyze the implementation effects of government subsidies. The model is constructed as follows:

$$lnTFP_{it}=\alpha_{0}+\alpha_{1}lnSub\_rd_{it-1}+\alpha_{2}lnSub\_cf_{it-1}+\sum_{j=1}^{7}\;\delta_{j}\;X_{jit}+u_{i}+v_{t}+\varepsilon_{it},$$ (6)

where i is the enterprise, t is the year, lnSub_rd_it-1_ is the logarithm of R&D subsidies lagged by one period, and lnSub_cf_it-1_ is the logarithm of production subsidies lagged by one period. Other variables are the same as in model (1). Table 3 presents the results.

**Table 3:** Effect of government subsidy lag period on TFP.

	lnTFP	
Variables	(1)	(2)
lnSub_rd_t-1_	0.004**	
	(2.11)	
lnSub_cf_t-1_		0.006***
		(4.07)
Covariates	Yes	Yes
Constant	0.922***	0.971***
	(9.84)	(10.67)
Firm-fixed effect	Yes	Yes
Time-fixed effect	Yes	Yes
R^2^	0.3712	0.3070
Observations	6823	6786

Note: t statistics are reported in parentheses; *** p<0.01,

** p<0.05,

* p<0.1.

Columns (1) and (2) in Table 3 show the regression results for the effect of government R&D and production subsidies lagged by one period on private enterprises’ TFP. The results show that the coefficients of the effect of R&D and production subsidies on enterprises’ TFP are 0.004 and 0.006, respectively. They pass the significance test with significance levels of 5% and 1%, respectively. Compared with the results in Table 2, the differences between the coefficients are very small, indicating that R&D and production subsidies can significantly increase private enterprises’ TFP, whether in the current period or lagged by one period. Next, we test the heterogeneity effect and the specific mechanisms of R&D and production subsidies’ effects on private enterprises’ TFP.

### 4.4 Heterogeneity analysis

#### 4.4.1 Industry heterogeneity

The sensitivity of different factor-intensive industries to government subsidies may vary. Following previous research, we divided industries into labor-intensive, capital-intensive, and technology-intensive industries [54]. S2 Table presents the classification method, and Table 4 presents the analysis results.

**Table 4:** Estimated effects of government subsidies on TFP of different industry groups.

	lnTFP					
Variables	Labor-intensive		Capital intensive		Technology-intensive	
	(1)	(2)	(3)	(4)	(5)	(6)
lnSub_rd	0.002		0.003		0.011***	
	(0.98)		(1.44)		(5.91)	
lnSub_cf		0.003		0.005*		0.008***
		(1.43)		(1.69)		(3.93)
Covariates	Yes	Yes	Yes	Yes	Yes	Yes
Constant	0.495	0.508	1.011***	1.065***	0.936***	1.019***
	(1.38)	(1.41)	(9.57)	(7.92)	(11.99)	(13.00)
Firm-fixed effect	Yes	Yes	Yes	Yes	Yes	Yes
Time-fixed effect	Yes	Yes	Yes	Yes	Yes	Yes
R^2^	0.182	0.193	0.310	0.207	0.141	0.172
Observations	1554	1570	1726	1726	4315	4337

Note: t statistics are reported in parentheses; *** p<0.01,

** p<0.05,

* p<0.1.

As shown in columns (1) and (3) of Table 4, the coefficients of R&D subsidies on labor-intensive and capital-intensive industries are 0.002 and 0.003, respectively, but the effect is not statistically significant. The coefficients in column (5) suggest that R&D subsidies have a positive effect on TFP for technology-intensive industries. The coefficients in columns (2), (4), and (6) are the effect of production subsidies on private enterprises’ TFP between different industries, and the coefficients are 0.003, 0.005, and 0.008, respectively. It is worth noting that the results for labor-intensive industries did not pass the significance test, while the significance levels of capital-intensive and technology-intensive industries were 10% and 1%, respectively. This indicates that production subsidies have no effect on labor-intensive industries and have the largest effect on technology-intensive industries. The results for capital-intensive industries are similar to those for labor-intensive industries, but the results for production subsidies are significant at the 10% level. Both R&D and production subsidies have the greatest effects on technology-intensive industries. In addition, R&D subsidies have a greater effect on the TFP of technology-intensive industries than production subsidies.

#### 4.4.2 Scale heterogeneity

There may be differences in the sensitivity of private enterprises of different sizes to government subsidies. Therefore, we examined the effect of government subsidies on private enterprises’ TFP by dividing the sample into scales. Following the “Classification of National Economic Industries (2017)” and the “Law of the People’s Republic of China on the Promotion of Small and Medium-Sized Enterprises,” we divided enterprise scale into three types: large, medium, and small. Table 5 presents the results. The coefficients in columns (1), (3), and (5) are the effects of R&D subsidies on the TFP of differently sized enterprises. The effect coefficients of large and medium-sized enterprises are 0.003 and 0.006, respectively; they are at least below the 10% significance level. Further analysis shows that the effect of government R&D subsidies on small enterprises does not pass the significance test. It is widely known that government R&D subsidies tend to have the greatest incentivizing effects on the TFP of medium-sized private enterprises. As shown in columns (2), (4), and (6), the effect of production subsidies on the TFP of large and small enterprises passes the significance test. However, the coefficient of medium-sized private enterprises is not significant. Moreover, we found that government production subsidies have a more significant promotion effect on the TFP of small enterprises. Overall, R&D subsidies have the most obvious effects on medium-sized enterprises’ TFP, while production subsidies have the greatest effect on small enterprises’ TFP. Specifically, medium-sized enterprises have a certain degree of financial strength. They can efficiently use R&D subsidy funds to improve TFP. Meanwhile, since small enterprises mainly face financing problems, production subsidies can help alleviate financing difficulties and increase TFP through scale effects.

**Table 5:** Estimated effects of government subsidies on TFP at different enterprise scales.

	lnTFP					
Variables	large enterprise		Med enterprise		Microenterprise	
	(1)	(2)	(3)	(4)	(5)	(6)
lnSub_rd	0.003***		0.006*		0.004	
	(3.15)		(1.93)		(0.79)	
lnSub_cf		0.004***		0.0004		0.005*
		(4.30)		(0.18)		(1.72)
Covariates	Yes	Yes	Yes	Yes	Yes	Yes
Constant	1.004***	1.008***	1.049***	1.019***	0.886***	1.008***
	(19.73)	(19.72)	(8.45)	(7.66)	(3.34)	(4.06)
Firm-fixed effect	Yes	Yes	Yes	Yes	Yes	Yes
Time-fixed effect	Yes	Yes	Yes	Yes	Yes	Yes
R^2^	0.446	0.447	0.209	0.183	0.135	0.109
Observations	4817	4862	1058	1053	1720	1718

Note: t statistics are reported in parentheses; *** p<0.01,

** p<0.05,

* p<0.1.

### 4.5 Further analysis and discussion

Here, we examine the mechanisms of the effects of different subsides on TFP and deal with the endogeneity issue.

#### 4.5.1 Mechanism of R&D subsidies’ effect on TFP

In this section, we test the mechanism of subsidies on TFP. The results are presented in Tables 6 and 7.

**Table 6:** Test for technological innovation mechanism in R&D subsidies’ effect on TFP.

	lnTFP			
Variables	(1)	(2)	(3)	(4)
lnRD	0.049***	0.050***		
	(8.24)	(8.08)		
lnSub_rd*lnRD		0.003**		
		(2.79)		
lnPatent			0.012***	0.009***
			(5.82)	(4.57)
lnSub_rd*lnPatent				0.001**
				(3.33)
Covariates	Yes	Yes	Yes	Yes
Constant	0.877***	0.850***	0.859***	0.920***
	(10.55)	(10.25)	(8.41)	(10.20)
Firm-fixed effect	Yes	Yes	Yes	Yes
Time-fixed effect	Yes	Yes	Yes	Yes
R^2^	0.405	0.367	0.211	0.373
Observations	7862	7594	6158	5990

Note: t statistics are reported in parentheses; *** p<0.01,

** p<0.05,

* p<0.1.

**Table 7:** Test for financing constraint mechanism in production subsidies’ effect on TFP.

	lnTFP	
Variables	(1)	(2)
CF	0.154***	0.141***
	(4.02)	(3.76)
lnSub_cf*CF		-0.002***
		(-5.70)
Covariates	Yes	Yes
Constant	1.094***	1.116***
	(11.55)	(11.84)
Firm-fixed effect	Yes	Yes
Time-fixed effect	Yes	Yes
R^2^	0.382	0.383
Observations	7863	7633

Note: t statistics are reported in parentheses; *** p<0.01,

** p<0.05,

* p<0.1.

As shown in column (1) of Table 6, the effect of innovation input on private enterprises’ TFP is positively correlated at the 1% level. The coefficient of the interaction term between R&D subsidies and innovation input is 0.003 in column (2). This indicates that an innovation input mechanism exists between R&D subsidies and enterprises’ TFP. The results in column (3) of Table 6 show that increasing the level of enterprise innovation output is conducive to increasing private enterprises’ TFP. From column (4), we can see that the coefficient of the interaction term between R&D subsidies and innovation output is significantly positive. This indicates that an innovation output mechanism exists between R&D subsidies and private enterprises’ TFP.

This study’s findings are contrary to those of Bernini and Pellegrini [11]. This could be attributable to differences in government subsidies and the measurement of TFP. Our results are, however, consistent with those of many other studies. Hsieh and Klenow [3], for example, also found that government subsidies can improve enterprises’ TFP by stimulating their enthusiasm for technological innovation. Sissoko [5] likewise noted that government subsidies can reduce enterprise innovation costs and risks, thereby increasing TFP. Carboni [24] also found that government subsidies can significantly promote enterprise R&D innovation and improve productivity. However, it should be noted that our results are more significant than those of previous studies. On the one hand, we studied the effect of R&D subsidies on enterprises’ TFP, and the incentive effect of R&D subsidies on enterprises’ R&D investment and technological innovation was found to be significant. On the other hand, improve the level of technological innovation, thereby making R&D subsidies more effective for promoting TFP. In summary, regardless of the indicators used to measure enterprises’ technological innovation, R&D subsidies can significantly improve private enterprises’ innovation levels, which in turn significantly promotes TFP, thus verifying H1.

We tested the financing constraint mechanism of production subsidies on TFP, as shown in Table 7. The coefficient in column (1) is 0.154, and the significance level is 1%. Since SA is a negative value, the larger the SA, the better the financing constraints of the enterprise. Therefore, a smaller financing pressure has a positive effect on private enterprises’ TFP. The coefficient of the interaction term between production subsidies and financing constraints is −0.002 in column (2), which is significant at the 1% level. As before, our conclusions are consistent with those of previous studies. Bernini and Cerqua [35] found that government subsidies can reduce the financing pressures of enterprises, reduce financing difficulty, and then increase TFP. Wang and Ai [40] suggested that government subsidies can provide price compensation for enterprises, reduce production and operation costs, and ultimately improve TFP. Furthermore, Wu [39] noted that government subsidies can broaden the financing channels of enterprises through implicit guarantees, reduce financing pressure, expand the scale of production and operations, and improve TFP. A production subsidy is a type of subsidy provided by the government to relieve enterprises’ financing pressures and help them open up the market. Additionally, in China, private enterprises face more prominent financing problems. Production subsidies can therefore help alleviate financing constraints for private enterprises, improve their operating efficiency, and ultimately improve their TFP. The results in Table 7 indicate that production subsidies can effectively alleviate the financing constraints of enterprises and increase their TFP, thus verifying H2.

#### 4.5.2 Endogeneity test

Private enterprises’ TFP may be affected by many factors; thus, endogeneity problems caused by missing variables may exist in the model. In addition, there could be a two-way causal relationship between government subsidies and the TFP of private enterprises, which might also lead to endogeneity. We aimed to find many instrumental variables. For example, the average value of different subsidies in industries other than the company’s industry was used as an instrumental variable, but it did not pass the correlation and exogeneity tests. These tests are necessary conditions for suitable instrumental variables. Regrettably, we did not find a suitable instrumental variable to solve the endogeneity problem. Therefore, the instrumental variable estimation method is not suitable for this study. However, difference GMM and system GMM are common approaches used to deal with endogeneity problems in many studies [55–59]. Difference GMM can solve the deviation of the endogeneity problem to the estimation result to a certain extent, but it cannot observe the characteristics of individual heterogeneity, and there are seriously weak instrumental variables. However, system GMM can overcome the weaknesses of difference GMM and alleviate the problem of weak instrumental variables. Therefore, we used both difference GMM and system GMM to solve the endogeneity problem. Table 8 presents the results.

**Table 8:** Results for subsidies’ effects on TFP after dealing with endogeneity.

	Difference GMM		System GMM	
Variables	(1)	(2)	(3)	(4)
L.lnTFP	0.394***	0.396***	0.760***	0.799***
	(2.89)	(2.88)	(13.11)	(12.14)
lnSub_rd	0.006*		0.010***	
	(1.90)		(3.43)	
lnSub_cf		0.005		0.007***
		(1.58)		(2.67)
Covariates	Yes	Yes	Yes	Yes
Constant			0.245**	0.190*
			(2.38)	(1.75)
Firm-fixed effect	Yes	Yes	Yes	Yes
Time-fixed effect	Yes	Yes	Yes	Yes
AR(1)	−1.65*	−1.58	−6.35***	−6.39***
AR(2)	−0.93	−1.06	−0.72	−0.95
Sargan Statistics	337.62***	320.12***	448.94***	484.78***
Number of instruments	47	47	69	69
Observations	4953	4863	5843	5805

Note: z statistics are reported in parentheses; *** p<0.01,

** p<0.05,

* p<0.1.

It can be seen from Table 8 that the effect of government subsidies on the TFP of private enterprises is consistent with the previous empirical results, again supporting H1. Moreover, the lag period of private enterprises’ TFP has a positive effect on the TFP of the current period, indicating that the improvement of enterprises’ TFP is a continuous process. The models in Table 8 pass the relevant tests of AR (2) and Sargan statistics, indicating that there is no overidentification problem, and the regression results are not affected by the second-order sequence. Thus, the differential GMM and system GMM methods are effective for this study, indicating that the estimated results of the model are reliable.

### 4.6 Robustness check

We conducted several additional regressions to check the robustness of our results. We changed the calculation method for TFP. S3 Table presents the results. Then, we verified the mechanism of different subsidies’ effects on TFP based on the mediating effect model. The results are shown in S4 and S5 Tables.

#### 4.6.1 Changing the measurement of TFP

Here, we use wages as a labor input to calculate TFP. As shown in S3 Table, the coefficients of R&D and production subsidies are 0.004 and 0.008, respectively, and both are significant at the 1% level. When R&D and production subsidies are increased by 1%, private enterprises’ TFP will increase by 0.004% and 0.008%, respectively. This shows that R&D subsidies and production subsidies play a significant role in promoting private enterprises’ TFP, which means that the estimated results are robust.

#### 4.6.2 Robustness test of the mechanism of government subsidies’ effect on TFP

First, we introduce the mediation effect model to test the internal mechanism of R&D subsidies’ effects on TFP. Taking innovation inputs as mechanism variables, the model is constructed as follows:

$$lnTFP_{it}=\alpha_{0}+\alpha_{1}lnSub\_rd_{it}+\sum_{j=1}^{7}\;\delta_{j}\;X_{jit}+u_{i}+v_{t}+\varepsilon_{it},$$ (7)

$$Z_{it}=\beta_{0}+\beta_{1}lnSub\_rd_{it}+\sum_{j=1}^{7}\;\delta_{j}\;X_{jit}+u_{i}+v_{t}+\varepsilon_{it},$$ (8)

$$lnTFP_{it}=\gamma_{0}+\gamma_{1}lnRD_{it}+\gamma_{2}lnSub\_rd_{it}+\sum_{j=1}^{7}\;\gamma_{j}\;X_{jit}+u_{i}+v_{t}+\varepsilon_{it},$$ (9)

where Z_it_ represents the mechanism variable of innovation input. The meanings of the covariates and subscripts are the same as those of model (1). The coefficient α_1_ in model (7) is the effect of R&D subsidies on private enterprises’ TFP. The coefficient β_1_ in model (8) is the effect of R&D subsidies on the mechanism variable. The coefficient γ_2_ in model (9) is the influence of the independent variables on TFP after controlling for the mechanism variables. Moreover, the coefficient γ_1_ is the effect of the mechanism variables on TFP after controlling for the influence of R&D subsidies.

From column (2) of S4 Table, we can see that there is a positive relationship between the R&D subsidy and innovation input. In column (3), the coefficients of R&D subsidies and innovation inputs on TFP are 0.003 and 0.049, respectively, and both are significant at the 1% level. It can be considered that R&D subsidies increase TFP by stimulating private enterprises to increase investment in innovation. The mediating effect of innovation input exists and is part of the mediation.

Second, we use the same model to check the robustness of the mediating effect of innovation output. The results are reported in columns (4), (5), and (6) of S4 Table. As shown in column (5), the number of firms granted patents increases by 0.033% when R&D subsidies increase by 1%. In column (6), the coefficient of lnPatent is 0.011 and significant at the 1% level. In addition, the coefficients of lnSub_rd are smaller when compared with column (4) and significant at the 1% level. Thus, we can conclude that innovation output exists between R&D subsidies and TFP, and innovation output is also part of the mediation.

Finally, we use financing constraints as an intermediary variable to test whether there is a financing constraint mechanism between production subsidies and TFP. The results are presented in S5 Table. Production subsidies can effectively alleviate the financing constraints of private enterprises. By incorporating production subsidies and financing constraints into the model, we found that both production subsidies and financing constraints have a significant positive effect on private enterprises’ TFP. This indicates that production subsidies can effectively improve private enterprises’ TFP by alleviating financing constraints. Therefore, the intermediary effect of financing constraints exists and is part of the mediation.

## 5. Conclusions and policy implications

In this study, we used data on Chinese A-share private listed manufacturing companies from 2009 to 2017 for analysis. The following conclusions can be drawn. First, both R&D subsidies and production subsidies have a positive effect on private enterprises’ TFP, with both passing endogeneity and robustness tests. Moreover, R&D and production subsidies lagged by one period can also significantly increase private enterprises’ TFP. Second, the effect of different subsidies on private enterprises’ TFP varies among different industries. Both R&D subsidies and production subsidies have a greater effect on private enterprises’ TFP in technology-intensive industries compared to other industries. Moreover, the effect of R&D subsidies on TFP in technology-intensive industries is greater than that of production subsidies. Third, government subsidies have different effects on enterprises of different sizes. R&D subsidies most obviously increase the productivity of medium-sized enterprises. Production subsidies have the greatest effect on promoting the productivity of small enterprises. Finally, although R&D and production subsidies play a significant role in promoting private enterprises’ TFP, their mechanisms are different. R&D subsidies can increase TFP by stimulating enterprises to increase R&D investment and innovation output; meanwhile, production subsidies can improve TFP by easing enterprises’ financing constraints and reducing their financial pressures.

Our findings have important implications for understanding how to implement subsidy policies. Empirical research shows that government subsidies are necessary. Therefore, China’s government should continue to increase subsidies to private enterprises to encourage them to innovate and increase their TFP. However, the effects of R&D and production subsidies vary among different industries and at different scales. Based on our results, the government should increase R&D subsidies for technology-intensive industries and medium-sized enterprises. For labor-intensive industries, capital-intensive industries, and small enterprises, it is necessary to increase production subsidies to ease financing constraints in the development of private enterprises. In addition, private enterprises must make full use of subsidy policies to improve their innovation and investment efficiency and ultimately improve their TFP.

This study has several limitations. First, due to a lack of data, we only used listed manufacturing companies as the analysis sample, which might produce biased estimates. Since listed companies represent high-quality companies in the same industry, government subsidies are more likely to have a positive effect on their TFP. In the future, it will be necessary to investigate the effect of subsidies on TFP based on different types of firm data. Second, we failed to find a suitable instrumental variable to deal with endogeneity. Hence, we used difference GMM and system GMM to solve the endogeneity problem; thus, our solution for the endogeneity problem might be imperfect. Future studies can improve our understanding of subsidies’ effects on TFP by finding a suitable instrumental variable to deal with endogeneity. In summary, in the future, more types of data and better solutions for endogeneity problems need to be explored to provide additional insights.

## Supporting information

S1 Table The classification method of government subsidy. (DOCX)

S2 Table The Classification method of industry. (DOCX)

S3 Table Robustness test of the effect of government subsidies on TFP. Note: t statistics are reported in parentheses; *** p<0.01, ** p<0.05, * p<0.1.
(DOCX)

S4 Table Robustness test of the innovation mechanism. Note: t statistics are reported in parentheses; *** p<0.01, ** p<0.05, * p<0.1.
(DOCX)

S5 Table Robustness test of the financing restraint mechanism. Note: t statistics are reported in parentheses; *** p<0.01, ** p<0.05, * p<0.1.
(DOCX)

S1 Dataset Dataset used in this study. (ZIP)
