# Supplementary material for: The effect of different government subsidies on total-factor productivity: Evidence from private listed manufacturing enterprises in China
Source: PLoS One. 2022 Jan 31;17(1):e0263018. doi: 10.1371/journal.pone.0263018 (PMC8803163; doi:10.1371/journal.pone.0263018)
Supplement: S1 Table — (DOCX) [file pone.0263018.s001.docx]

**S2 Table. The classification method of government subsidy.**

| **The type of government subsidy** | **Government subsidy content** |
| --- | --- |
| Production subsidy | Subsidies containing the following keywords: major investment, investment promotion, stable subsidies, unemployment insurance, employment subsidies, production base construction, production and development funds, loans, export support, value-added tax return, tax reduction tax rebate, enterprise support, import Passes, market development fund subsidies, financing guarantee subsidies, fiscal activities, etc. |
| R&D subsidy | Subsidies containing the following keywords: new product development, technical transformation, independent innovation special bonus, scientific and technological achievements transformation, patent reward, scientific and technological reward, brand reward, science, and technology project, innovation support, well-known trademark, two fusion, intellectual property rewards, Issue, production, information, information construction, topics, research and development projects, talent subsidies, high-tech enterprises, small giants, “863” talent programs, torch plans, etc. |
