# Supplementary material for: The effect of different government subsidies on total-factor productivity: Evidence from private listed manufacturing enterprises in China
Source: PLoS One. 2022 Jan 31;17(1):e0263018. doi: 10.1371/journal.pone.0263018 (PMC8803163; doi:10.1371/journal.pone.0263018)
Supplement: S2 Table — (DOCX) [file pone.0263018.s002.docx]

**S3 Table. The classification method of industry.**

| **The type of industry** | **Industry segmentation** |
| --- | --- |
| Labor intensive | Textile and clothing, clothing; textile industry; non-metallic mineral products; metal products; furniture manufacturing; wood processing and wood, bamboo, vine, brown, grass products industry; agricultural and sideline food processing industry; leather, fur, feathers, and Its products and footwear industry; food manufacturing; cultural education, work and beauty, sports and entertainment products manufacturing; rubber and plastic products; printing and recording medium copies. |
| Capital intensive | The black metal smelting and calendaring plus industry; chemical fiber manufacturing; chemical raw materials and chemical manufacturing; wine, beverage and refining tea manufacturing; petroleum processing, coking, and nuclear fuel processing industry; universal equipment manufacturing; non-ferrous metal smelting and calendaring Industry; paper and paper products; other manufacturing. |
| Technical intensive | Electrical machinery and equipment manufacturing; computer, communication, and other electronic equipment manufacturing; railway, ship, aerospace, and other transport equipment manufacturing; medical manufacturing; instrumentation manufacturing; special equipment manufacturing; automotive manufacturing; waste resources Comprehensive utilization. |
