# Supplementary material for: The effect of different government subsidies on total-factor productivity: Evidence from private listed manufacturing enterprises in China
Source: PLoS One. 2022 Jan 31;17(1):e0263018. doi: 10.1371/journal.pone.0263018 (PMC8803163; doi:10.1371/journal.pone.0263018)
Supplement: S3 Table — Note: t statistics are reported in parentheses; *** p<0.01, ** p<0.05, * p<0.1. (DOCX) [file pone.0263018.s003.docx]

**S4 Table. Robustness test of the effect of government subsidies on TFP.**

| **Variables** | **lnTFP** | |
| --- | --- | --- |
|  | **(1)** | **(2)** |
| lnSub_rd | 0.004^***^ |  |
|  | (3.82) |  |
| lnSub_cf |  | 0.008^***^ |
|  |  | (5.97) |
| Covariates | Yes | Yes |
| Constant | 1.070^***^ | 1.120^***^ |
|  | (16.79) | (16.88) |
| Firm-fixed effect | Yes | Yes |
| Time-fixed effect | Yes | Yes |
| R^2^ | 0.336 | 0.321 |
| Observations | 7596 | 7634 |

Note: t statistics are reported in parentheses; *** p<0.01, ** p<0.05, * p<0.1.
