# Supplementary material for: The effect of different government subsidies on total-factor productivity: Evidence from private listed manufacturing enterprises in China
Source: PLoS One. 2022 Jan 31;17(1):e0263018. doi: 10.1371/journal.pone.0263018 (PMC8803163; doi:10.1371/journal.pone.0263018)
Supplement: S4 Table — Note: t statistics are reported in parentheses; *** p<0.01, ** p<0.05, * p<0.1. (DOCX) [file pone.0263018.s004.docx]

**S5 Table. Robustness test of the innovation mechanism.**

| **Variables** | **The mechanism of innovation input** | | | **The mechanism of innovation output** | | |
| --- | --- | --- | --- | --- | --- | --- |
|  | **lnTFP** | **lnRD** | **lnTFP** | **lnTFP** | **lnPatent** | **lnTFP** |
|  | **(1)** | **(2)** | **(3)** | **(4)** | **(5)** | **(6)** |
| lnSub_rd | 0.005*** | 0.041*** | 0.003*** | 0.005*** | 0.033*** | 0.002*** |
|  | (4.22) | (4.64) | (2.88) | (4.22) | (2.77) | (3.59) |
| lnRD |  |  | 0.049*** |  |  |  |
|  |  |  | (8.06) |  |  |  |
| lnPatent |  |  |  |  |  | 0.011*** |
|  |  |  |  |  |  | (5.33) |
| Covariates | Yes | Yes | Yes | Yes | Yes | Yes |
| Constant | 0.875*** | 0.365 | 0.856*** | 0.875*** | 0.233 | 0.909*** |
|  | (10.22) | (1.00) | (10.60) | (10.22) | (0.27) | (10.21) |
| Firm-fixed effect | Yes | Yes | Yes | Yes | Yes | Yes |
| Time-fixed effect | Yes | Yes | Yes | Yes | Yes | Yes |
| R^2^ | 0.364 | 0.202 | 0.364 | 0.364 | 0.127 | 0.355 |
| Observations | 7595 | 7596 | 7594 | 7595 | 5991 | 5990 |

Note: t statistics are reported in parentheses; *** p<0.01, ** p<0.05, * p<0.1.
