# Supplementary material for: The effect of different government subsidies on total-factor productivity: Evidence from private listed manufacturing enterprises in China
Source: PLoS One. 2022 Jan 31;17(1):e0263018. doi: 10.1371/journal.pone.0263018 (PMC8803163; doi:10.1371/journal.pone.0263018)
Supplement: S5 Table — Note: t statistics are reported in parentheses; *** p<0.01, ** p<0.05, * p<0.1. (DOCX) [file pone.0263018.s005.docx]

**S6 Table. Robustness test of the financing restraint mechanism.**

| **Variables** | **lnTFP** | **CF** | **lnTFP** |
| --- | --- | --- | --- |
|  | **(1)** | **(2)** | **(3)** |
| lnSub_cf | 0.008^***^ | 0.006^***^ | 0.006^***^ |
|  | (5.91) | (5.96) | (5.48) |
| CF |  |  | 0.142^***^ |
|  |  |  | (3.79) |
| Covariates | Yes | Yes | Yes |
| Constant | 0.932^***^ | −1.297^***^ | 1.116^***^ |
|  | (10.47) | (−18.79) | (11.87) |
| Firm-fixed effect | Yes | Yes | Yes |
| Time-fixed effect | Yes | Yes | Yes |
| R^2^ | 0.373 | 0.859 | 0.385 |
| Observations | 7633 | 7636 | 7633 |

Note: t statistics are reported in parentheses; *** p<0.01, ** p<0.05, * p<0.1.
